# Predicting Spontaneous Termination of Atrial Fibrillation Based on Analysis of Standard Electrocardiograms: A Systematic Review

**DOI:** 10.1111/anec.70025

**Published:** 2024-10-25

**Authors:** Brandon Wadforth, Jing Soong Goh, Kathryn Tiver, Sobhan Salari Shahrbabaki, Ivaylo Tonchev, Dhani Dharmaprani, Anand N. Ganesan

**Affiliations:** ^1^ College of Medicine and Public Health Flinders University Adelaide Australia; ^2^ Division of Medicine, Cardiac and Critical Care Flinders Medical Centre Adelaide Australia; ^3^ Department of Cardiac Electrophysiology Flinders Medical Centre Adelaide Australia; ^4^ Australian Institute for Machine Learning University of Adelaide Adelaide Australia

**Keywords:** atrial fibrillation, electrocardiogram, entropy, frequency analysis, machine learning, prediction, termination, time–frequency analysis

## Abstract

**Background:**

Forward prediction of atrial fibrillation (AF) termination is a challenging technical problem of increasing significance due to rising AF presentations to emergency departments worldwide. The ability to non‐invasively predict which AF episodes will terminate has important implications in terms of clinical decision‐making surrounding treatment and admission, with subsequent impacts on hospital capacity and the economic cost of AF hospitalizations.

**Methods and Results:**

MEDLINE, EMCare, CINAHL, CENTRAL, and SCOPUS were searched on 29 July 2023 for articles where an attempt to predict AF termination was made using standard surface ECG recordings. The final review included 35 articles. Signal processing techniques fit into three broad categories including machine learning (*n* = 14), entropy analysis (*n* = 12), and time–frequency/frequency analysis (*n* = 9). Retrospectively processed ECG data was used in all studies with no prospective validation studies. Most studies (*n* = 33) utilized the same ECG database, which included recordings that either terminated within 1 min or continued for over 1 h. There was no significant difference in accuracy between groups (H(2) = 0.058, *p*‐value = 0.971). Only one study assessed recordings earlier than several minutes preceding termination, achieving 92% accuracy using the central 10 s of paroxysmal episodes lasting up to 174.

**Conclusions:**

No studies attempted to forward predict AF termination in real‐time, representing an opportunity for novel prospective validation studies. Multiple signal processing techniques have proven accurate in predicting AF termination utilizing ECG recordings sourced from a database retrospectively.

## Graphical abstract

Three main approaches to predicting the spontaneous termination of atrial fibrillation using standard surface electrocardiograms included machine learning, entropy analysis, and time–frequency/frequency analysis. The median accuracy was 90% with no significant differences between groups (H(2) = 0.058, *p*‐value = 0.971). No studies validated their prediction models prospectively.

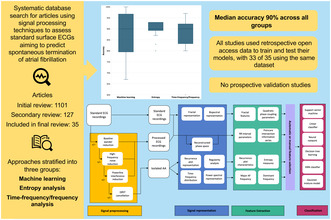

## 1 Introduction

Atrial fibrillation (AF) is the most common arrhythmia and has a rising incidence (Hindricks et al. 2021; Joglar et al. 2024). Therefore, emergency department presentations with recent‐onset AF are expected to increase with time. Although electrical cardioversion is highly successful in terminating recent‐onset AF, a substantial proportion of these patients will revert to a sinus rhythm spontaneously. A recent trial found delayed cardioversion to be non‐inferior to early cardioversion for patients with recent‐onset AF (within 36 h), with 69% of patients spontaneously reverting to sinus rhythm within 48 h (Pluymaekers et al. 2019). Here, avoiding electrical cardioversion reduced procedural risk and time spent in the emergency department. However, delaying cardioversion for every patient presenting with recent‐onset AF will postpone treatment for those who require it. Therefore, the ability to non‐invasively predict AF termination would be a useful clinical tool to guide decision‐making around treatment and disposition of patients. If accurate, this could facilitate early discharge strategies for patients likely to spontaneously terminate, thereby reducing patient load on emergency departments and unnecessary hospital admissions. To this point, however, clinicians have only had clinical information to inform the likelihood of spontaneous termination of AF. A systematic review identified short duration of AF (< 48 h), absence of prior heart disease, low number of prior AF episodes, and normal atrial dimensions as predictors of spontaneous AF termination (Pluymaekers et al. 2020). Notably, there was substantial variability between studies, limiting the applicability of these findings and therefore an improved way to stratify patients presenting to emergency departments with recent‐onset AF if needed.

There have been previous attempts to predict the spontaneous termination of AF using a standard surface 12‐lead electrocardiogram (ECG) recording (Cantini et al. 2004; Roberts and Povinelli 2004; Chiarugi et al. 2007; Saberi et al. 2008; Mohebbi and Ghassemian 2014; Mainardi, Matteucci, and Sassi 2004; Sun and Wang 2008, 2009; Sezgin 2013; Lemay et al. 2004; Parvaneh et al. 2012; Esgiar and Chakravorty 2004; Logan and Healey 2004; Bukkapatnam et al. 2008; Alcaraz and Rieta 2007a, 2009a, 2009b, 2010, 2007b, 2008, 2012a, 2012b; Vaya and Rieta 2007, 2008, 2009; Alcaraz et al. 2006; Vaya et al. 2006; Langley et al. 2004; Mora et al. 2004; Petrutiu et al. 2004; Xi and Shkurovich 2004; Hayn et al. 2004; Hayn, Kollmann, and Schreier 2007; Nilsson et al. 2004, 2006). To our knowledge, there has been no systematic review of these studies. Given the perceived benefits of predicting AF termination, we sought to review the current literature to determine the timepoint of prediction, sample size, data source, signal analysis techniques, and accuracy described in published studies. This could then be used to inform future prospective studies aiming to predict AF termination in real‐time with the end goal to offering a streamlined and optimized management pathway for these patients.

## 2 Methods

Our study protocol and objectives were predetermined and registered with PROSPERO (CRD42023450230). We searched for articles which aimed to predict termination of AF using a standard surface ECG. On 29 July 2023, a systematic search of multiple databases, including MEDLINE, EMCare, CINAHL, CENTRAL, and SCOPUS was performed, and results were downloaded into a Covidence library. The search strategy was designed and conducted in consultation with a senior librarian, and the search terms are outlined in the Data S1. Articles were limited to English only. Additional articles were found through manual bibliography searches. Articles were excluded if there was no attempt to classify standard surface ECG recordings of AF in humans as either terminating or non‐terminating, and therefore the accuracy of their approach could not be assessed and compared with others. Additionally, secondary sources were excluded. Studies that explored termination without treatment or with antiarrhythmic medications were included, yet studies where termination was achieved through electrical cardioversion and ablation were excluded. Three reviewers (B.W., JS.G., and A.N.G.) were involved in the appraisal of articles, and disagreements regarding their suitability were resolved via consensus.

Approaches were classified into three groups, machine learning, entropy analysis, and time–frequency/frequency analysis based on the predominant signal processing approach described in their methodology, acknowledging that there was overlap between groups. The accuracy, defined by the percentage of correctly classified episodes in a testing environment, was compared among groups that used the same dataset to allow a fair comparison of methods. The normality assumption was assessed using a Shapiro–Wilk's test, and subsequently, differences in accuracy were assessed using a Kruskal‐Wallis test. Statistical analyses were performed using R version 4.4.0.

## 3 Results

**FIGURE 1:**
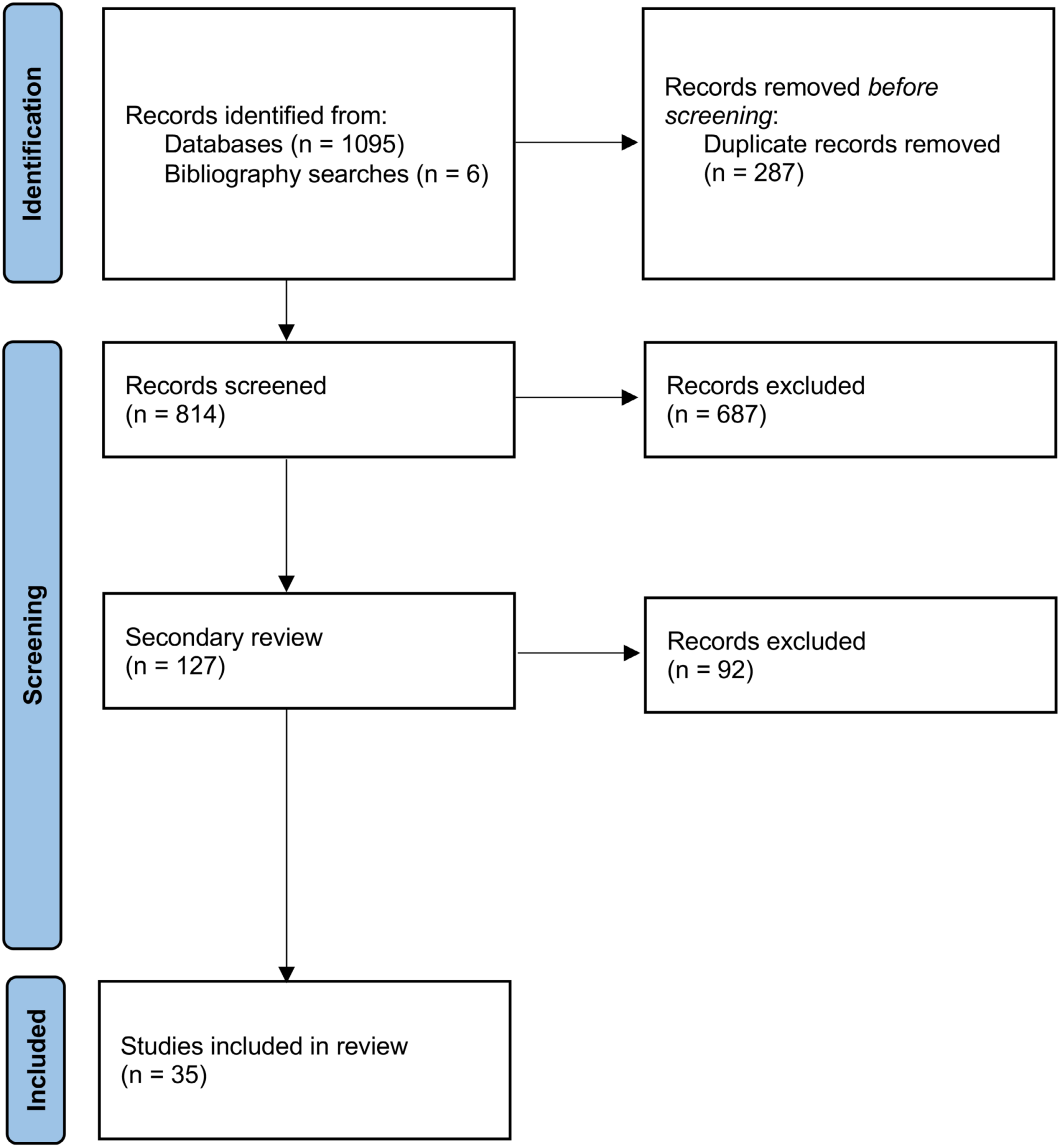
Search flow diagram for studies included in this review. Databases included MEDLINE, EMCare, CINAHL, CENTRAL, and SCOPUS.

Database searches revealed 1095 articles, of which 287 were duplicates. Six articles were subsequently included from hand‐searching references. There were 687 articles excluded on initial screening (Figure 1), leaving 127 selected for detailed secondary review. Of these, 35 were ultimately retained in the final analysis (Cantini et al. 2004; Roberts and Povinelli 2004; Chiarugi et al. 2007; Saberi et al. 2008; Mohebbi and Ghassemian 2014; Mainardi, Matteucci, and Sassi 2004; Sun and Wang 2008, 2009; Sezgin 2013; Lemay et al. 2004; Parvaneh et al. 2012; Esgiar and Chakravorty 2004; Logan and Healey 2004; Bukkapatnam et al. 2008; Alcaraz and Rieta 2007a, 2009a, 2009b, 2010, 2007b, 2008, 2012a, 2012b; Vaya and Rieta 2007, 2008, 2009; Alcaraz et al. 2006; Vaya et al. 2006; Langley et al. 2004; Mora et al. 2004; Petrutiu et al. 2004; Xi and Shkurovich 2004; Hayn et al. 2004; Hayn, Kollmann, and Schreier 2007; Nilsson et al. 2004, 2006).

### 3.1 Sample Size and Data Source

All studies validated their model's performance using retrospective recordings, with no studies attempting to predict termination in real‐time using a prospective cohort. Most studies (*n* = 33) used the AF termination database (AFT‐DB) exclusively to train and test their models. This dataset was made publicly available by Physionet for the Computers in Cardiology Challenge 2004 (Moody 2004). The recordings included leads II and V1 with a sampling rate of 128 Hz and 16‐bit resolution. The training set included 10 recordings for groups S, T, and N, which either terminated within 1 min, terminated immediately, or did not terminate for at least 1 h after the recording, respectively. Test set A included 30 T or N recordings. Test set B included T or S recordings and therefore was not relevant for this review. Notably, one study (Alcaraz and Rieta 2012b) merged the training set and test sets, and another (Sezgin 2013) combined both test sets A and B.

One study (Hayn, Kollmann, and Schreier 2007) developed their model with the AFT‐DB then validated it on multiple retrospective datasets, including the AFT‐DB, the MIT‐BIH AF database (AF‐DB), and the MIT‐BIH arrhythmia database (ARR‐DB). The 2 min preceding termination were used for the AF‐DB which contained 25 recordings at a sampling frequency of 250 Hz, and the ARR‐DB, which contained 50 recordings with a sampling frequency of 360 Hz.

Only one study used recordings earlier than 2 min preceding termination (Alcaraz and Rieta 2010). In this study, Alcaraz and Rieta (2010) sourced Holter recordings of 61 patients presenting to cardiologists with new‐onset AF and processed it retrospectively. They included the central 10 s of the longest AF episodes for patients with paroxysmal AF (*n* = 29) against randomly chosen 10 s intervals for patients with persistent AF (*n* = 31).

### 3.2 Signal Preprocessing

Signal preprocessing was commonly performed. Many groups (*n* = 18) specifically mentioned addressing baseline wander using bidirectional high‐pass filtering with the cut‐off frequency ranging from 0.25 to 1 Hz (Saberi et al. 2008; Sun and Wang 2008; Lemay et al. 2004; Parvaneh et al. 2012; Bukkapatnam et al. 2008; Alcaraz and Rieta 2007a, 2009a, 2009b, 2010, 2007b, 2008, 2012a, 2012b; Vaya and Rieta 2008, 2009; Petrutiu et al. 2004; Xi and Shkurovich 2004; Nilsson et al. 2006). Some (*n* = 16) then applied a low‐pass filter, generally a Butterworth or IIR Chebyshev filter, with a cut‐off frequency ranging from 30 to 70 Hz (Saberi et al. 2008; Sun and Wang 2008; Bukkapatnam et al. 2008; Alcaraz and Rieta 2007a, 2009a, 2009b, 2010, 2007b, 2008, 2012a, 2012b; Vaya and Rieta 2008, 2009; Petrutiu et al. 2004; Xi and Shkurovich 2004; Nilsson et al. 2006). Several groups then increased the sampling rate from 128 to 1054 Hz using cubic spline interpolation. Most studies (*n* = 30) utilized QRST cancelation to isolate the AA (Cantini et al. 2004; Roberts and Povinelli 2004; Chiarugi et al. 2007; Saberi et al. 2008; Mohebbi and Ghassemian 2014; Mainardi, Matteucci, and Sassi 2004; Sun and Wang 2008; Lemay et al. 2004; Bukkapatnam et al. 2008; Alcaraz and Rieta 2007a, 2009a, 2009b, 2010, 2007b, 2008, 2012a, 2012b; Vaya and Rieta 2007, 2008, 2009; Alcaraz et al. 2006; Vaya et al. 2006; Langley et al. 2004; Mora et al. 2004; Petrutiu et al. 2004; Xi and Shkurovich 2004; Hayn et al. 2004; Hayn, Kollmann, and Schreier 2007; Nilsson et al. 2004, 2006), typically using the average beat subtraction (ABS) method. Further processing was occasionally undergone to remove ventricular residue from the AA. For instance, one group (Hayn, Kollmann, and Schreier 2007) used cubic spline interpolation within a 55 ms window of the QRS complexes to reduce ventricular noise after QRST cancelation. Other approaches to QRST cancelation included spatiotemporal cancelation (Nilsson et al. 2004, 2006), principal component analysis (Mora et al. 2004), and bandpass filtering (Roberts and Povinelli 2004).

### 3.3 Signal Processing Approaches and Accuracy

**FIGURE 2:**
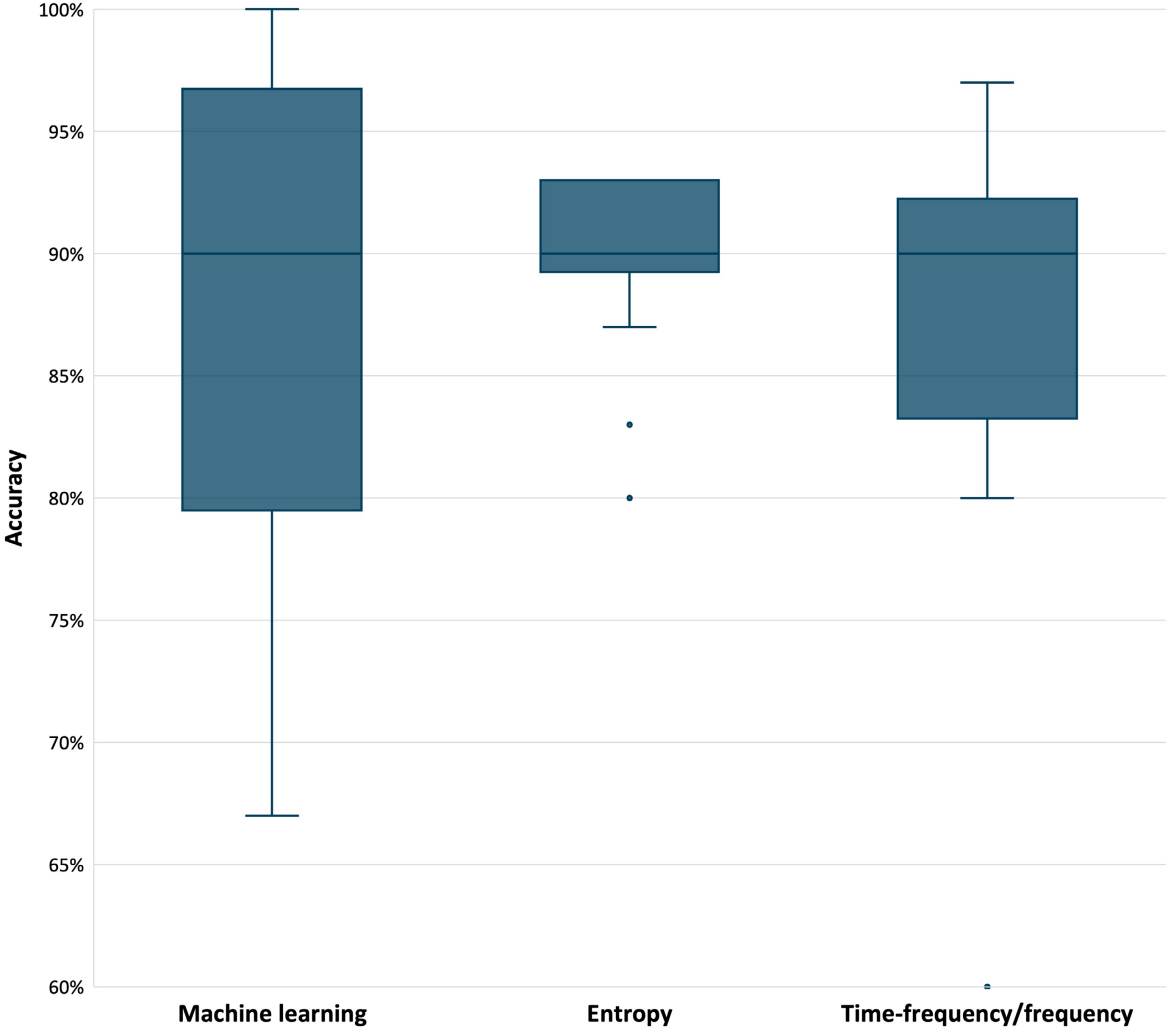
Accuracy among categories. Box and whisker plot indicating the median (90% for all groups) and interquartile ranges.

In total, 31 studies reported their model's accuracy when specifically applied to the 30 testing sets of ECG signals in the AFT‐DB, which allowed direct comparison of methods. The remaining studies were not compared here as they either used a different dataset to test their model (Alcaraz and Rieta 2010; Hayn, Kollmann, and Schreier 2007) or reported accuracy in both the training set and testing set as a combined value (Sezgin 2013; Alcaraz and Rieta 2012b). There was no significant difference in the accuracy among all compared groups (H(2) = 0.058, *p*‐value = 0.971), with the median being 90.0 for all three (machine learning IQR = 0.77, 0.97; entropy analysis IQR = 0.89, 0.93; time–frequency/frequency analysis IQR = 0.87, 0.92), as shown in Figure 2.

#### 3.3.1 Machine Learning

**FIGURE 3:**
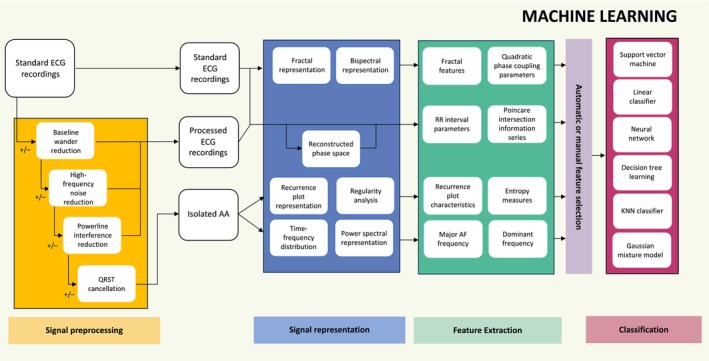
Machine learning process. Signal preprocessing was variably applied to raw ECG signals to reduce baseline wander, reduce high‐ and low‐frequency noise and/or cancel QRST complexes to isolate the atrial activity (AA). Some groups did not require any signal preprocessing. Signal representation is the process of presenting ECG data or isolated AA into an alternative domain, for example transitioning from the time domain into the frequency domain. This allows extraction of variables which cannot be ascertained from the raw data. Feature extraction is the process of extracting numeric variables from the processed signals for subsequent analysis. Feature selection was either automatic whereby a statistical process was utilized to optimize selection of features to be fed into the machine learning classifier or manual, whereby the authors selected the features themselves. Classification is the stage where previously selected features were fed into a machine learning algorithm which categorized the sample as terminating or non‐terminating.

There were 13 studies within this group. Machine learning approaches generally entailed several stages, which could include signal preprocessing, signal representation, feature extraction, feature selection, and classification as depicted in Figure 3. Classification approaches included linear classifiers (Cantini et al. 2004; Roberts and Povinelli 2004; Chiarugi et al. 2007; Saberi et al. 2008; Mohebbi and Ghassemian 2014), neural networks (Mainardi, Matteucci, and Sassi 2004; Sun and Wang 2008; Sezgin 2013), support vector machines (Lemay et al. 2004; Sun and Wang 2009; Parvaneh et al. 2012), a KNN classifier (Esgiar and Chakravorty 2004), a Gaussian mixture model (Logan and Healey 2004), and decision tree learning (Bukkapatnam et al. 2008). Signal representation was generally time–frequency or frequency analysis using a Fourier or wavelet transform (Cantini et al. 2004; Chiarugi et al. 2007; Saberi et al. 2008; Mohebbi and Ghassemian 2014; Mainardi, Matteucci, and Sassi 2004; Lemay et al. 2004; Bukkapatnam et al. 2008), although some groups used other methods. Extracted features included spectral parameters, fractal features, AA characteristics, and RR interval parameters, including heart rate variability. Six groups utilized automatic feature selection such as sequential forward search algorithms (Sun and Wang 2008, 2009), discriminant analysis (Chiarugi et al. 2007; Mohebbi and Ghassemian 2014), covariance matrices (Saberi et al. 2008), or a genetic algorithm (Parvaneh et al. 2012) to optimize the data fed into the classifier. The remaining groups (Cantini et al. 2004; Roberts and Povinelli 2004; Mainardi, Matteucci, and Sassi 2004; Sezgin 2013; Lemay et al. 2004; Esgiar and Chakravorty 2004; Logan and Healey 2004; Bukkapatnam et al. 2008) fed all extracted features into the classifier.

Maximum accuracy was achieved using a machine learning approach with either a fuzzy support vector machine (Sun and Wang 2009) or linear discriminant analysis (LDA) (Saberi et al. 2008) as indicated in Table 1. Sun and colleagues (Sun and Wang 2009) first processed the signals to obtain 11 statistical features from the RR interval, such as the RR interval mean or RR interval skewness, and additionally obtained geometric features of the RR interval from a Poincare plot. Five features were selected using a sequential forward search algorithm with a Davies‐Bouldin criterion. A fuzzy support vector machine then used these features to classify recordings with 100% accuracy. Saberi et al. (2008) preprocessed their signals and applied QRST cancelation. Feature extraction entailed frequency analysis via Welch's method, analysis of the RR interval (of samples prior to QRST cancelation), and time–frequency analysis using a short‐time Fourier transform (STFT). Feature selection was achieved using a covariance matrix with a scattering criterion. Two features, the delta RR and main peak frequency, were then chosen for classification with LDA achieving 100% accuracy.

**TABLE 1:** Approach to classification and accuracy of included studies.

			Sample size and data source			Signal processing approach and accuracy		
Ref	First Author	Year	Data source	Sample size	Minutes preceding termination	Signal analysis approach	Subgroup	Accuracy (%)
5	Cantini	2004	AFT‐DB	30	1 min	ML	Linear classifier	90
6	Roberts	2004	AFT‐DB	30	1 min	ML	Linear classifier	67
7	Chiarugi	2007	AFT‐DB	30	1 min	ML	Linear classifier	90
8	Saberi	2008	AFT‐DB	30	1 min	ML	Linear classifier	100
9	Mohebbi	2014	AFT‐DB	30	1 min	ML	Linear classifier	97
10	Mainardi	2004	AFT‐DB	30	1 min	ML	Neural network	87
11	Sun	2008	AFT‐DB	30	1 min	ML	Neural network	97
12	Sezgin	2013	AFT‐DB	80^a^	1 min	ML	Neural network	96^a^
13	Lemay	2004	AFT‐DB	30	1 min	ML	Support vector machine	67
14	Sun	2009	AFT‐DB	30	1 min	ML	Support vector machine	100
15	Parvaneh	2012	AFT‐DB	30	1 min	ML	Support vector machine	87
16	Esgiar	2004	AFT‐DB	30	1 min	ML	KNN classifier	73
17	Logan	2004	AFT‐DB	30	1 min	ML	Gaussian mixture model	77
18	Bukkapatnam	2008	AFT‐DB	30	1 min	ML	Decision tree learning	93
19	Alcaraz	2007	AFT‐DB	30	1 min	E	SampEn of MAW	90
20	Alcaraz	2009	AFT‐DB	30	1 min	E	SampEn of MAW	90
21	Alcaraz	2009	AFT‐DB	30	1 min	E	SampEn of MAW	90
22	Alcaraz	2010	Independently sourced	61	Variable^b^	E	SampEn of MAW	92
23	Vaya	2007	AFT‐DB	30	1 min	E	SampEn of SP	80
24	Vaya	2008	AFT‐DB	30	1 min	E	SampEn of SP with LDA	87
25	Vaya	2009	AFT‐DB	30	1 min	E	SampEn of SP with LDA	93
26	Alcaraz	2007	AFT‐DB	30	1 min	E	SampEn of wavelet parameters	93
27	Alcaraz	2008	AFT‐DB	30	1 min	E	SampEn of wavelet parameters	93
28	Alcaraz	2006	AFT‐DB	30	1 min	E	SampEn of wavelet parameters	90
29	Alcaraz	2012	AFT‐DB	30	1 min	E	Wavelet entropy	93
30	Vaya	2006	AFT‐DB	30	1 min	E	ApEn of SP	83
31	Langley	2004	AFT‐DB	30	1 min	TF/F	DF	80
32	Mora	2004	AFT‐DB	30	1 min	TF/F	DF	90
33	Petrutiu	2004	AFT‐DB	30	1 min	TF/F	DF	97
34	Xi	2004	AFT‐DB	30	1 min	TF/F	DF	83
35	Hayn	2004	AFT‐DB	30	1 min	TF/F	Major AF frequency	93
36	Hayn	2007	AFT‐DB, AF‐DB, ARR‐DB	75	2 min	TF/F	Major AF frequency	< 60
37	Nilsson	2004	AFT‐DB	30	1 min	TF/F	Major AF frequency	90
38	Nilsson	2006	AFT‐DB	30	1 min	TF/F	Major AF frequency	90
39	Alcaraz	2012	AFT‐DB	50	1 min	TF/F	Wavelet transform and CTM	96

Abbreviations: AFT‐DB, atrial fibrillation termination database; CTM, central tendency measure; DF, dominant frequency; E, entropy analysis; LDA, linear discriminant analysis; MAW, main atrial wave; ML, machine learning; SP, spectral parameters; TF/F, time–frequency/frequency analysis.

^a^ All groups (S, T and N) merged for analysis rather than just comparing T and N recordings.

^b^ Central 10 s interval of longest duration AF episode on Holter recording (episode range 12–174 min).

#### 3.3.2 Entropy Analysis

**FIGURE 4:**
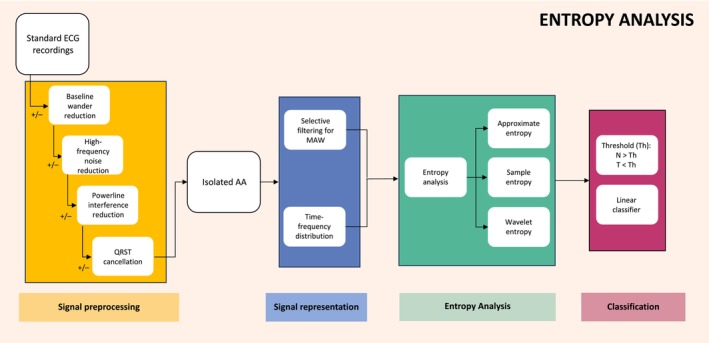
Entropy analysis process. All entropy studies underwent signal preprocessing to produce isolated atrial activity (AA) which could be subjected to entropy analysis. Signal representation included either creating a time–frequency distribution via the use of a transform function or selective filtering of the AA around the dominant frequency to create a graphical representation of the so‐called main atrial wave (MAW). Entropy analysis was then done whereby the approximate, sample or wavelet entropy of the signal was calculated. Classification was then achieved using a threshold whereby signals with entropy above a specific point were classified as non‐terminating and signals below were classified as terminating or using a linear classifier.

Entropy analysis aims to describe the amount of randomness within a signal. Entropy‐based classification was used in 12 studies, with accuracies ranging from 80% to 93%. Of these, four studies assessed the sample entropy of the main atrial wave (MAW) (Alcaraz and Rieta 2007a, 2009a, 2009b, 2010), three assessed the sample entropy of spectral parameters (Vaya and Rieta 2007, 2008, 2009), with two then further assessing with discriminant analysis (Vaya and Rieta 2008, 2009), three used sample entropy of reconstructed wavelet coefficient vectors (Alcaraz and Rieta 2007b, 2008; Alcaraz et al. 2006), one used wavelet entropy (Alcaraz and Rieta 2012a), and one used the approximate entropy of spectral parameters (Vaya et al. 2006) (Figure 4). The accuracy of these approaches was generally similar. The MAW was calculated by identifying the dominant frequency (DF) within a 3–9 Hz range using a fast Fourier transform, then applying selective filtering to the AA trace around the DF (Alcaraz and Rieta 2010). Spectral parameters were obtained using transform functions. Wavelet coefficient vectors were obtained using a wavelet transform function, and these were reconstructed into the time domain prior to entropy analysis (Alcaraz et al. 2006). Optimal thresholds were usually selected using receiver operating characteristic curves.

#### 3.3.3 Time‐Frequency/Frequency Analysis

**FIGURE 5:**
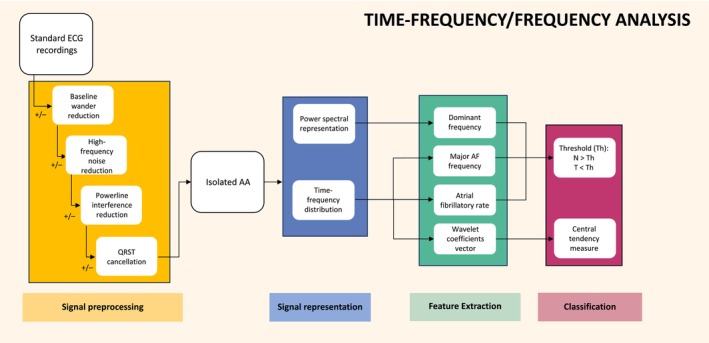
Time–frequency/frequency analysis process. All time–frequency/frequency analysis studies underwent signal preprocessing to produce isolated atrial activity (AA). Signal representation included either creating a time–frequency distribution or power spectral representation via the use of a transform functions. Features were then extracted from these signals including dominant frequency, major atrial fibrillation frequency, atrial fibrillatory rate and wavelet coefficients vectors. All signals were then classified using a threshold whereby signals with entropy above a specific point were classified as non‐terminating and signals below were classified as terminating however the wavelet coefficients vectors were classified using a central tendency measure.

There were nine studies that featured classification based solely on time–frequency or frequency information (Figure 5). Eight of these studies used the AFT‐DB, with accuracies ranging from 80% to 96%. Four classified recordings using a simple cut‐off for the DF within the 3–10 Hz range obtained via Fast Fourier Transform or Welch's method (Langley et al. 2004; Mora et al. 2004; Petrutiu et al. 2004; Xi and Shkurovich 2004). The calculated optimal DF used was 4.9, 5.5, or 5.7 Hz depending on the study. Two studies (Nilsson et al. 2004, 2006) used a simple 5.7 Hz cut‐off for the major AF frequency, which was obtained via STFT. Hayn et al. (2004) and Hayn, Kollmann, and Schreier (2007) created a predictive formula containing the major AF frequency, the mean RR interval, and an empirical coefficient to classify recordings. They achieved 93% accuracy with this method on the AFT‐DB; however, their model's performance reduced to < 60% accuracy when tested on the AF‐DB and ARR‐DB (Hayn, Kollmann, and Schreier 2007). Alcaraz and Rieta (2012b) used a wavelet transform in combination with a central tendency measure, achieving 96% accuracy.

## 4 Discussion

Many patients presenting with new‐onset AF to emergency departments spontaneously terminate, making decisions around which patients to cardiovert difficult. Recent evidence has found that a watch‐and‐wait approach with delayed cardioversion is non‐inferior to early cardioversion in patients presenting with recent‐onset AF (Pluymaekers et al. 2019). This strategy avoids procedural risks however also delays treatment for approximately a third of patients who do not spontaneously terminate (Pluymaekers et al. 2019). Forward prediction of AF termination would therefore offer significant clinical benefit if it could be achieved in real‐time for patients presenting to hospitals. It would facilitate early discharge for patients likely to terminate and early referral for cardioversion for patients unlikely to spontaneously revert to sinus rhythm. Though, to the best of our knowledge, there have been no attempts to forward predict AF termination prospectively in patients presenting with recent‐onset AF.

### 4.1 Data Source

We identified 35 studies that aimed to predict AF termination using retrospective surface ECG recordings. Studies generally used the AFT‐DB, which had a limited time point of prediction with the T group signals terminating after just 1 min. Although this time point of prediction would not be useful clinically, the signal analysis approaches from these studies can inform future attempts. Most groups validated their model using recordings from the same dataset they trained their model with. This likely produces a false elevation in accuracy due to model overadaptation to a single data source. Only one study (Hayn, Kollmann, and Schreier 2007) trained their model using the AFT‐DB, then subsequently validated it on other databases. Although they were able to achieve 93% accuracy on the AFT‐DB test set, their accuracy in the AF‐DB and ARR‐DB was < 60%, indicating poor generalisability of their model. This therefore questions the applicability of most of the studies to ECG recordings other than the AFT‐DB given the lack of model validation beyond this dataset. Another study (Alcaraz and Rieta 2010), however, described an approach to predicting AF termination utilizing recordings external to the AFT‐DB with a reasonable accuracy of 92%, suggesting potential viability in a prospective validation study.

### 4.2 Approach to Signal Preprocessing

Most recordings were processed prior to feature extraction. Signal preprocessing generally included the application of filtering operations to reduce baseline wander and high‐frequency noise, with some further processing to reduce powerline interference. This approach assumes that signal and noise exist in different frequency bands. Given ECG recordings obtained in the clinical setting are likely to contain more noise than those individually selected for the Physionet challenge database, these signal preprocessing steps are likely to be of importance in attempts to prospectively classify non‐terminating and terminating AF episodes.

Multiple methods of QRST cancelation were used including ABS, spatiotemporal cancelation, bandpass filtering, and principal component analysis. Most groups used the single‐lead ABS QRST cancelation method, which involved establishing the average morphology of QRS and T waves and then subtracting this template from the detected QRS and T waves. Notably, spatiotemporal cancelation has previously been shown to be superior to ABS in extracting ventricular signal as this method additionally accounts for respiratory‐induced beat‐to‐beat variability in QRST morphology (Stridh and Sörnmo 2001). Spatiotemporal cancelation, however, is dependent on multiple leads to extract spatial information, yet most studies in this review had limited access to just leads II and V1. Whilst QRST cancelation could therefore theoretically be improved in future research, the magnitude of its effect on classification accuracy is not clear.

### 4.3 Approach to Signal Classification

Studies were classified into three general groups that achieved similar outcomes. It remains difficult to extrapolate the utility of any given approach for use in a prospective validation study given the sample homogeneity among the current literature. We have therefore avoided quantitative meta‐analysis of our findings and focused on qualitative evaluation of key findings.

#### 4.3.1 Machine Learning

Most machine learning groups extracted frequency or time–frequency information obtained following QRST cancelation. Alternatively, some machine learning groups (Sezgin 2013; Sun and Wang 2009; Parvaneh et al. 2012; Esgiar and Chakravorty 2004; Logan and Healey 2004) avoided the use of QRST cancelation by using other features such as RR intervals. Six groups used automatic feature selection (Chiarugi et al. 2007; Saberi et al. 2008; Mohebbi and Ghassemian 2014; Sun and Wang 2008, 2009; Parvaneh et al. 2012) rather than feeding every feature into the classifier, and these tended to have superior accuracy, suggesting this step could be of importance.

Two machine learning studies achieved 100% accuracy (Saberi et al. 2008; Sun and Wang 2009). Saberi and colleagues (Saberi et al. 2008) used both RR interval parameters in addition to power spectral information obtained post QRST cancelation. Alternatively, the features generated by Sun and Wang (2009) did not require QRST cancelation. Both used automatic feature selection. Saberi et al. (2008) fed their features into an LDA classifier, while Sun and Wang (2009) fed their features into a fuzzy support vector machine. These two studies have shown that optimal results can be achieved through extracting multiple features from the ECG followed by automatic feature selection prior to feeding these data into the classifier. This should however be interpreted with caution due to the limited sizes of datasets used in these studies, as well as the absence of prospective validation studies.

Some articles compared multiple methods of machine learning in their analysis. Bukkapatnam et al. (2008) tested the performance of statistical analysis of variance, a neural network, an adaptive neurofuzzy interface system, and a classification and regression tree (CART) model in their publication. Although the CART model performed best in their study, neural networks are known to perform better with access to larger datasets and therefore may prove superior in a different setting. Langley et al. (2004) compared fibrillatory frequency with LDA and an artificial neural network, finding that the simple fibrillatory frequency cut‐off method was the most effective. Again, the performance of machine learning may have been suppressed due to the small sample size. Machine learning algorithms can also become oversensitive when trained on a single data source. Therefore, it remains unclear whether machine learning would improve with larger datasets or alternatively worsen, as the current literature may have models that were oversensitive to the AFT‐DB and may not perform as well in another setting.

#### 4.3.2 Entropy Analysis

Entropy has multiple applications in AF, including detection, characteristic determination, and mapping (Dharmaprani et al. 2018). Here, entropy analysis has been shown to accurately predict AF termination using surface ECG recordings. Entropy is derived from information theory and aims to quantify the amount of uncertainty or randomness within a signal such that increased entropy is associated with increased randomness (Dharmaprani et al. 2018). Theoretically, more wandering wavelets throughout atrial tissue would produce a more disorganized electrical signal, and therefore one would expect higher entropy in the AA for these patients along with a lower likelihood of spontaneous termination (Alcaraz and Rieta 2007a). The accuracy of studies using entropy were generally similar ranging from 83% to 93%; however, those applying entropy following power spectral analysis rather than time–frequency analysis tended to achieve inferior results. Additionally, accuracy was improved when more advanced approaches to classification were used rather than thresholds, as shown by Vaya and Rieta (2009). With sample entropy alone, they were able to achieve an accuracy of 87% in predicting termination with the AFT‐DB, which was subsequently increased to 93% when they incorporated discriminant analysis to classify recordings. Therefore, while entropy has a strong theoretical basis for its use in predicting AF termination and has proven to be accurate, predictive performance would likely be improved with the addition of machine learning following entropy analysis.

#### 4.3.3 Time‐Frequency/Frequency Analysis

Time–frequency/frequency analysis of the surface ECG for patients with AF was independently proposed by two groups in 1998 based on the relationship between frequency and atrial refractoriness (Holm et al. 1998; Bollmann et al. 1998). DF signifies the activation rate and therefore refractoriness of the highest amplitude atrial signal, such that lower frequency signals represent longer refractoriness (Alcaraz and Rieta 2009c; Latchamsetty and Kocheril 2009). Atrial refractoriness is a key determinant of the atrial wavelength, which is associated with the size and number of wavefronts propagating through the atria (Bollmann et al. 1998). Therefore, an increased DF would theoretically be expected in patients with more wavefronts and thus more disorganized electrical activity, which would be less likely to spontaneously terminate. Bollmann et al. (1999) have tested this theory and shown that persistent AF (by definition, non‐terminating AF) is indeed correlated with higher frequency fibrillation. Further, studies on the antiarrhythmic drug bepridil showed that reduced DF following administration was associated with AF termination (Fujiki et al. 2003, 2004). Time–frequency analysis can indicate the major AF frequency, or alternatively the atrial fibrillatory rate (AFR), where the frequency is multiplied by a factor of 60 to describe the frequency in fibrillations per minute. Like DF, the major AF frequency and AFR indicate atrial refractoriness; however, here the averaged refractoriness over time is represented (Bollmann et al. 2006). Similar to Bollmann's reported correlation of high‐frequency fibrillation with persistent AF, Choudhary et al. (2013) have demonstrated that high AFR is associated with non‐terminating AF. The relevance of AFR in terminating AF has further support in the literature through drug studies, with some publications demonstrating that larger decreases in AFR are correlated with AF termination (Aunes‐Jansson et al. 2013; Schwartz and Langberg 2000); however, this was not consistent across all studies (Mochalina et al. 2015).

In addition to its strong theoretical foundation, frequency analysis provides a computationally easy method to differentiate recordings, although the results achieved with frequency analysis were suboptimal compared to other methods when applied to the AFT‐DB. Indeed, one group (Petrutiu et al. 2004) was able to achieve 97% accuracy using a DF cut‐off, yet their classification depended on comparison of spectral information from the ultimate and penultimate second prior to termination, which would not be clinically useful. Time–frequency analysis delivered improved accuracy. A wavelet transform was used in conjunction with a central tendency measure delivering 96% accuracy (Alcaraz and Rieta 2012b). More commonly, the major AF frequency was used, which represents the averaged DF over time (Hayn et al. 2004; Hayn, Kollmann, and Schreier 2007; Nilsson et al. 2004, 2006). This measure provided good accuracy when validated on the AFT‐DB test set; however, performance greatly reduced when challenged with another database, as previously mentioned (Hayn, Kollmann, and Schreier 2007).

#### 4.3.4 Limitations

There was significant sample homogeneity among the literature, with 33 of 35 studies using ECG recordings from the same database, which is a major limitation to understanding how the three approaches to signal processing would perform when challenged with real patient data. Therefore, we currently cannot extrapolate with confidence how each method would perform when applied to datasets varied by their size, the timing of the ECG recordings, and the degree of noise. Further, the overlap in signal processing methodology between groups limits our ability to distinguish algorithm performance. Additionally, no study prospectively validated their model or attempted to forward predict termination in patients presenting to emergency departments. This therefore limits the applicability of these findings in addressing the clinical problem of determining which patients presenting to emergency departments with recent‐onset AF would be best suited to early versus delayed cardioversion. However, our findings could guide future research by providing insight into potential approaches to this problem.

#### 4.3.5 Areas for Future Research

A question that remains unanswered from the included studies is whether ECGs taken at initial presentation could be utilized to predict AF termination, as this would be the most clinically useful timepoint of prediction. A study by Husser et al. (2007) assessed this using ECGs taken from the time of presentation to examine features associated with termination. Their multivariate analysis revealed that the only independent predictor of termination was AFR, and they subsequently applied an AFR cut‐off of 355 fpm to those recordings, demonstrating that this approach would have produced an accuracy of 84%. These findings were further supported by Choudhary et al. (2013), who demonstrated that an AFR below 350 fpm was an independent predictor of spontaneous termination of AF within 18 h when assessing ECGs in 148 patients presenting to the emergency department with AF. Future research could assess the performance of machine learning applied to ECGs taken at the timepoint of emergency presentation, as some machine learning methods were able to achieve greater accuracy than time–frequency analysis in the AFT‐DB. Further, integrating entropy‐based features into machine learning classifiers may further enhance the accuracy, given that entropy analysis has demonstrated effectiveness in predicting termination hours prior (Alcaraz and Rieta 2010).

In addition to the above, there is scarce application in other datasets, and therefore validating the signal processing methods on larger datasets using real patient data is needed to progress this area. Further research could then be directed at the application in real‐time, ultimately aiming to guide clinical decision‐making. In addition, the described literature is focused solely on application of signal processing without concurrent use of clinical factors known to be associated with spontaneous termination of AF. These clinical factors include short duration of AF (< 48 h), absence of prior heart disease, low number of prior AF episodes, and normal atrial dimensions (Pluymaekers et al. 2020). A clear strength of machine learning is its ability to integrate multiple sources of information, which could include these clinical factors, and therefore the amalgamation of signal processing with clinical data in machine learning algorithms could be an area for future research.

## 5 Conclusions

The prediction of AF termination using ECG recordings has been achieved with good accuracy using retrospective data. Although maximal accuracy was only achieved using machine learning, we found no statistically significant difference between groups. A major limitation in the current literature is sample homogeneity, with a single ECG database being used in 33 of 35 studies. This database has a limited timepoint of prediction of just 1 min prior to termination; however, one study demonstrated the ability to predict termination hours prior. To date, there have been no attempts to validate model performance in real‐time using a prospective patient cohort, presenting an opportunity for future research.

## Author Contributions

B.W., A.N.G., and K.T. conceived and designed the research protocol. B.W. performed the database search. B.W. and J.S.G. reviewed articles for their suitability with A.N.G. resolving conflicts. B.W. performed statistical analysis. B.W., J.S.G., K.T., S.S.S., I.T., D.D., and A.N.G. provided key intellectual content and made critical contribution to manuscript revision.

## Conflicts of Interest

The authors declare no conflicts of interest.

## Supporting information


Data S1:

## Data Availability

Data sharing is not applicable to this article as no new data were created or analyzed in this study.
