# Supplementary material for: Predicting Spontaneous Termination of Atrial Fibrillation Based on Analysis of Standard Electrocardiograms: A Systematic Review
Source: Ann Noninvasive Electrocardiol. 2024 Oct 25;29(6):e70025. doi: 10.1111/anec.70025 (PMC11503732; doi:10.1111/anec.70025)
Supplement: Supplementary file 1 — Data S1: [file ANEC-29-e70025-s001.zip › PROSPERO_Prediction of the spontaneous termination of atrial fibrillation based on analysis of the standard electrocardiogram.pdf]

National Institute for Health Research

UNIVERSITY of York
Centre for Reviews and Dissemination

Systematic review

*Fields that have an **asterisk (*)** next to them means that they **must be answered. Word limits** are provided for each section. You will be unable to submit the form if the word limits are exceeded for any section. Registrant means the person filling out the form*.

This record cannot be edited because it has been marked as out of scope

1. * Review title. [1 change]

Give the title of the review in English

Prediction of the Spontaneous Termination of Atrial Fibrillation Based on Analysis of the Standard Electrocardiogram: A Systematic Review.

2. Original language title.

For reviews in languages other than English, give the title in the original language. This will be displayed with the English language title.

3. * Anticipated or actual start date.

Give the date the systematic review started or is expected to start.

01/10/2022

4. * Anticipated completion date.

Give the date by which the review is expected to be completed.

01/10/2024

5. * Stage of review at time of this submission. [1 change]

This field uses answers to initial screening questions. It cannot be edited until after registration.

Tick the boxes to show which review tasks have been started and which have been completed.

Update this field each time any amendments are made to a published record.

The review has not yet started: No

Review stage	Started	Completed
Preliminary searches	Yes	Yes
Piloting of the study selection process	Yes	Yes
Formal screening of search results against eligibility criteria	Yes	Yes
Data extraction	Yes	No
Risk of bias (quality) assessment	No	No
Data analysis	No	No

Provide any other relevant information about the stage of the review here.

6. * Named contact.

The named contact is the guarantor for the accuracy of the information in the register record. This may be any member of the review team.

Brandon Wadforth

Email salutation (e.g. "Dr Smith" or "Joanne") for correspondence:

Dr Wadforth

7. * Named contact email.

Give the electronic email address of the named contact.

brandon.wadforth@flinders.edu.au

8. Named contact address

Give the full institutional/organisational postal address for the named contact.

Sturt Rd, Bedford Park SA 5042

9. Named contact phone number.

Give the telephone number for the named contact, including international dialling code.

+61481156607

10. * Organisational affiliation of the review.

Full title of the organisational affiliations for this review and website address if available. This field may be completed as 'None' if the review is not affiliated to any organisation.

Flinders University

Organisation web address:

https://www.flinders.edu.au

11. * Review team members and their organisational affiliations. [1 change]

Give the personal details and the organisational affiliations of each member of the review team. Affiliation refers to groups or organisations to which review team members belong. **NOTE: email and country now MUST be entered for each person, unless you are amending a published record. PLEASE USE AN INSTITUTIONAL EMAIL ADDRESS IF POSSIBLE.**

Dr Brandon Wadforth. Flinders University
Professor Anand Ganesan. Flinders University
Dr Kathryn Tiver. Flinders University
Mr Jing Soong Goh. Flinders University
Dr Sobhan Salari Shahrbabaki. Flinders University

12. * Funding sources/sponsors.

Details of the individuals, organizations, groups, companies or other legal entities who have funded or sponsored the review.

Not applicable

Grant number(s)

State the funder, grant or award number and the date of award

13. * Conflicts of interest.

List actual or perceived conflicts of interest (financial or academic).

None

14. Collaborators.

Give the name and affiliation of any individuals or organisations who are working on the review but who are not listed as review team members. **NOTE: email and country must be completed for each person, unless you are amending a published record.**

15. * Review question.

State the review question(s) clearly and precisely. It may be appropriate to break very broad questions down into a series of related more specific questions. Questions may be framed or refined using PI(E)COS or similar where relevant.

The primary objective is to assess whether standard 12 lead Electrocardiograms (ECGs) are able to predict the successful termination of atrial fibrillation in a patient or not.

16. * Searches.

[1 change]

State the sources that will be searched (e.g. Medline). Give the search dates, and any restrictions (e.g. language or publication date). Do NOT enter the full search strategy (it may be provided as a link or attachment below.)

Studies were identified through systematic searches of the following databases

- the Cochrane Library (including the Cochrane Central Register of Controlled Trials (CENTRAL));

- MEDLINE via Ovid platform;

- EMcare via Ovid platform;

- Cumulative Index of Nursing and Allied Health Literature (CINAHL); and

- Scopus database.

The search strategy will be designed and conducted in consultation with a senior librarian. The search will be restricted to the English language articles only, with no restriction on publication dates.

We will conduct hand searches of the reference lists of included articles and citation tracking to identify relevant articles.

17. URL to search strategy.

[1 change]

Upload a file with your search strategy, or an example of a search strategy for a specific database, (including the keywords) in pdf or word format. In doing so you are consenting to the file being made publicly accessible. Or provide a URL or link to the strategy. Do NOT provide links to your search **results**.

https://www.crd.york.ac.uk/PROSPEROFILES/450230_STRATEGY_20231226.pdf

Alternatively, upload your search strategy to CRD in pdf format. Please note that by doing so you are consenting to the file being made publicly accessible.

Do not make this file publicly available until the review is complete

18. * Condition or domain being studied.

Give a short description of the disease, condition or healthcare domain being studied in your systematic review.

The applicability and use of standard 12 lead ECGs in predicting whether atrial fibrillation in a patient will successfully terminate or sustain.

19. * Participants/population.

Specify the participants or populations being studied in the review. The preferred format includes details of both inclusion and exclusion criteria.

Studies of humans identified as being in atrial fibrillation will be included.

20. * Intervention(s), exposure(s).

Give full and clear descriptions or definitions of the interventions or the exposures to be reviewed. The preferred format includes details of both inclusion and exclusion criteria.

Standard 12-lead ECG utilisation for indication of atrial fibrillation.

21. * Comparator(s)/control.

Where relevant, give details of the alternatives against which the intervention/exposure will be compared (e.g. another intervention or a non-exposed control group). The preferred format includes details of both inclusion and exclusion criteria.

Not applicable

22. * Types of study to be included.

Give details of the study designs (e.g. RCT) that are eligible for inclusion in the review. The preferred format includes both inclusion and exclusion criteria. If there are no restrictions on the types of study, this should be stated.

We will include randomised controlled trials, non-random assignment trial, full-text studies, conference abstracts, and unpublished data. There will be no exclusions based on sample size or country.

23. Context.

Give summary details of the setting or other relevant characteristics, which help define the inclusion or exclusion criteria.

Studies conducted in different settings including but not limited to hospitals, community care centres and private clinics will be included in this review.

24. * Main outcome(s). [1 change]

Give the pre-specified main (most important) outcomes of the review, including details of how the outcome is defined and measured and when these measurement are made, if these are part of the review inclusion criteria.

1) ECG analysis and identification of parameters capable of predicting either spontaneous termination of AF

Measures of effect

Please specify the effect measure(s) for you main outcome(s) e.g. relative risks, odds ratios, risk difference, and/or 'number needed to treat.

Nonlinear measures of ECGs.

25. * Additional outcome(s).

List the pre-specified additional outcomes of the review, with a similar level of detail to that required for main outcomes. Where there are no additional outcomes please state 'None' or 'Not applicable' as appropriate to the review

None

Measures of effect

Please specify the effect measure(s) for you additional outcome(s) e.g. relative risks, odds ratios, risk difference, and/or 'number needed to treat.

26. * Data extraction (selection and coding).

Describe how studies will be selected for inclusion. State what data will be extracted or obtained. State how this will be done and recorded.

One reviewer will perform the full search strategy, remove duplicates and screen article titles. Abstracts will be screened independently by two reviewers and included if they satisfy the inclusion criteria. Discrepancies will be discussed by the two reviewers with a third investigator until consensus is reached. Full-text copies will be obtained if studies appear to meet inclusion criteria or if it is unclear if they meet inclusion criteria. Full text articles will be independently reviewed by two investigators for inclusion. Any discrepancies will be discussed with a third investigator until consensus is achieved.

Data extraction will be undertaken independently by two investigators using a standardised data extraction tool or RevMan (Cochrane Collaboration). Once complete, the two investigators will compare data extracts. Discrepancies in extracted data will be discussed and resolved with a third investigator - if required.

27. * Risk of bias (quality) assessment.

State which characteristics of the studies will be assessed and/or any formal risk of bias/quality assessment tools that will be used.

Two reviewers will independently assess the risk of bias for included studies using a validated and published risk of bias tool if applicable to the study designs included in the review.

28. * Strategy for data synthesis.

Describe the methods you plan to use to synthesise data. This **must not be generic text** but should be **specific to your review** and describe how the proposed approach will be applied to your data. If meta-analysis is planned, describe the models to be used, methods to explore statistical heterogeneity, and software package to be used.

The reviewer will report a summary table of data from each included study.

The suitability for meta-analysis will be assessed by the review team after data extraction has been

undertaken. If appropriate, a meta-analysis will be undertaken.

A summary of findings will be presented descriptively for both research questions.

29. * Analysis of subgroups or subsets.

State any planned investigation of ‘subgroups’. Be clear and specific about which type of study or participant will be included in each group or covariate investigated. State the planned analytic approach.

None planned.

30. * Type and method of review.

Select the type of review, review method and health area from the lists below.

Type of review

Cost effectiveness

No

Diagnostic

No

Epidemiologic

No

Individual patient data (IPD) meta-analysis

No

Intervention

No

Living systematic review

No

Meta-analysis

No

Methodology

No

Narrative synthesis

No

Network meta-analysis

No

Pre-clinical

No

Prevention

No

Prognostic
No

Prospective meta-analysis (PMA)
No

Review of reviews
No

Service delivery
No

Synthesis of qualitative studies
No

Systematic review
Yes

Other
No

Health area of the review

Alcohol/substance misuse/abuse
No

Blood and immune system
No

Cancer
No

Cardiovascular
Yes

Care of the elderly
No

Child health
No

Complementary therapies
No

COVID-19
No

Crime and justice
No

Dental

No

Digestive system

No

Ear, nose and throat

No

Education

No

Endocrine and metabolic disorders

No

Eye disorders

No

General interest

No

Genetics

No

Health inequalities/health equity

No

Infections and infestations

No

International development

No

Mental health and behavioural conditions

No

Musculoskeletal

No

Neurological

No

Nursing

No

Obstetrics and gynaecology

No

Oral health

No

Palliative care

No

Perioperative care

No

Physiotherapy

No

Pregnancy and childbirth

No

Public health (including social determinants of health)

No

Rehabilitation

No

Respiratory disorders

No

Service delivery

No

Skin disorders

No

Social care

No

Surgery

No

Tropical Medicine

No

Urological

No

Wounds, injuries and accidents

No

Violence and abuse

No

31. Language.

Select each language individually to add it to the list below, use the bin icon to remove any added in error.
English

There is not an English language summary

32. * Country.

Select the country in which the review is being carried out. For multi-national collaborations select all the countries involved.

Australia

33. Other registration details.

Name any other organisation where the systematic review title or protocol is registered (e.g. Campbell, or The Joanna Briggs Institute) together with any unique identification number assigned by them. If extracted data will be stored and made available through a repository such as the Systematic Review Data Repository (SRDR), details and a link should be included here. If none, leave blank.

34. Reference and/or URL for published protocol.

If the protocol for this review is published provide details (authors, title and journal details, preferably in Vancouver format)

Add web link to the published protocol.

Or, upload your published protocol here in pdf format. Note that the upload will be publicly accessible.

No I do not make this file publicly available until the review is complete

Please note that the information required in the PROSPERO registration form must be completed in full even if access to a protocol is given.

35. Dissemination plans.

Do you intend to publish the review on completion?

Yes

Give brief details of plans for communicating review findings.?

36. Keywords.

Give words or phrases that best describe the review. Separate keywords with a semicolon or new line. Keywords help PROSPERO users find your review (keywords do not appear in the public record but are included in searches). Be as specific and precise as possible. Avoid acronyms and abbreviations unless these are in wide use.

ECG; Atrial Fibrillation; AF; Termination; Persistence

37. Details of any existing review of the same topic by the same authors.

If you are registering an update of an existing review give details of the earlier versions and include a full bibliographic reference, if available.

38. * Current review status.

Update review status when the review is completed and when it is published.New registrations must be ongoing so this field is not editable for initial submission.

Please provide anticipated publication date

Review_Ongoing

39. Any additional information.

Provide any other information relevant to the registration of this review.

40. Details of final report/publication(s) or preprints if available.

Leave empty until publication details are available OR you have a link to a preprint (NOTE: this field is not editable for initial submission). List authors, title and journal details preferably in Vancouver format.

Give the link to the published review or preprint.
