# Supplementary material for: Predicting Spontaneous Termination of Atrial Fibrillation Based on Analysis of Standard Electrocardiograms: A Systematic Review
Source: Ann Noninvasive Electrocardiol. 2024 Oct 25;29(6):e70025. doi: 10.1111/anec.70025 (PMC11503732; doi:10.1111/anec.70025)
Supplement: Supplementary file 1 — Data S1: [file ANEC-29-e70025-s001.zip › Search Strategy.docx]

Search Terms

| **#** | **Master Search** | **OVID** | **CENTRAL** | **CINAHL** | **SCOPUS** |
| --- | --- | --- | --- | --- | --- |
| 1 | Atrial Fibrillation/ | Atrial Fibrillation/ | ((atrial OR auricular OR atrium) NEAR/1 fibril*) | MH “Atrial Fibrillation” | TITLE-ABS-KEY ((atrial OR auricular OR atrium) W/1 fibril*) |
| 2 | ((atrial OR auricular OR atrium) ADJ fibril*) | ((atrial or auricular or atrium) adj fibril*).tw,kf. | (electrocardiog* OR ECG* OR EKG* OR (("12-lead" OR "12 lead") NEAR/1 (ECG OR EKG))) | TI OR AB ((atrial OR auricular OR atrium) N1 fibril*) | TITLE-ABS-KEY (electrocardiog* OR ECG* OR EKG* OR (("12-lead" OR "12 lead") W/1 (ECG OR EKG))) |
| 3 | 1 OR 2 | 1 or 2 | (terminat* OR rever* OR convert OR conversion*) | 1 OR 2 | TITLE-ABS-KEY (terminat* OR rever* OR convert OR conversion*) |
| 4 | Electrocardiography/ | Electrocardiography/ | (analys* OR predict* OR associated factor* OR parameter*) | MH “Electrocardiography” | TITLE-ABS-KEY (analys* OR predict* OR associated factor* OR parameter*) |
| 5 | (electrocardiog* OR ECG* OR EKG* OR (("12-lead" OR "12 lead") ADJ (ECG OR EKG))) | (electrocardiog* or ECG* or EKG* or (("12-lead" or "12 lead") adj (ECG or EKG))).tw,kf. | 1 AND 2 AND 3 AND 4 in Title Abstract Keyword | TI OR AB (electrocardiog* OR ECG* OR EKG* OR (("12-lead" OR "12 lead") N1 (ECG OR EKG))) | 1 AND 2 AND 3 AND 4 |
| 6 | 4 OR 5 | 4 or 5 |  | 4 OR 5 |  |
| 7 | (terminat* OR rever* OR convert OR conversion*) | (terminat* or rever* or convert or conversion*).tw,kf. |  | TI OR AB (terminat* OR rever* OR convert OR conversion*) |  |
| 8 | (analys* OR predict* OR associated factor* OR parameter*) | (analys* or predict* or associated factor* or parameter*).tw,kf. |  | TI OR AB (analys* OR predict* OR associated factor* OR parameter*) |  |
| 9 | 3 AND 6 AND 7 AND 8 | 3 and 6 and 7 and 8 |  | 3 AND 6 AND 7 AND 8 |  |
